# 1,25(OH)_2_D_3_ dependent overt hyperactivity phenotype in klotho-hypomorphic mice

**DOI:** 10.1038/srep24879

**Published:** 2016-04-25

**Authors:** Christina B. Leibrock, Jakob Voelkl, Makoto Kuro-o, Florian Lang, Undine E Lang

**Affiliations:** 1 Department of Physiology, Cardiology & Vascular Medicine, University of Tübingen, Gmelinstr. 5, 72076 Tübingen, Germany; 2 Center for Molecular Medicine, Jichi Medical University, 3311-1 Yakushiji, Shimotsuke, Tochigi 329-0498, Japan; 3 Department of Psychiatry, University of Basel, Wilhelm Klein-Strasse 27, CH-4012 Basel, Switzerland

## Abstract

Klotho, a protein mainly expressed in kidney and cerebral choroid plexus, is a powerful regulator of 1,25(OH)_2_D_3_ formation. Klotho-deficient mice (*kl/kl*) suffer from excessive plasma 1,25(OH)_2_D_3_-, Ca^2+^- and phosphate-concentrations, leading to severe soft tissue calcification and accelerated aging. NH_4_Cl treatment prevents tissue calcification and premature ageing without affecting 1,25(OH)_2_D_3_-formation. The present study explored the impact of excessive 1,25(OH)_2_D_3_ formation in NH_4_Cl-treated *kl/kl-*mice on behavior. To this end *kl/kl*-mice and wild-type mice were treated with NH_4_Cl and either control diet or vitamin D deficient diet (LVD). As a result, plasma 1,25(OH)_2_D_3_-, Ca^2+^- and phosphate-concentrations were significantly higher in untreated and in NH_4_Cl-treated *kl/kl-*mice than in wild-type mice, a difference abrogated by LVD. In each, open field, dark-light box, and O-maze NH_4_Cl-treated *kl/kl-*mice showed significantly higher exploratory behavior than untreated wild-type mice, a difference abrogated by LVD. The time of floating in the forced swimming test was significantly shorter in NH_4_Cl treated *kl/kl-*mice compared to untreated wild-type mice and to *kl/kl*-mice on LVD. In wild-type animals, NH_4_Cl treatment did not significantly alter 1,25(OH)_2_D_3_, calcium and phosphate concentrations or exploratory behavior. In conclusion, the excessive 1,25(OH)_2_D_3_ formation in klotho-hypomorphic mice has a profound effect on murine behavior.

Klotho is expressed mainly in the kidney, but is highly expressed as well in choroid plexus of the brain^1^. The extracellular domain of the transmembrane protein may be cleaved off and enter blood or cerebrospinal fluid^1^. Klotho is a powerful inhibitor of 1α-25-hydroxyvitamin D hydroxylase (1 α hydroxylase) thus preventing 1,25-dihydroxyvitamin D_3_ (1,25(OH)_2_D_3_) formation^1^. Klotho influences mineral metabolism in addition by up-regulation of Ca^2+^ channels^2^ and down-regulation of phosphate transport^3^,^4^. Klotho affects further channels and transport proteins including Na^+^/K^+^-ATPase^5^,^6^, Na^+^/Ca^2+^-exchanger^7^, Ca^2+^ channels^8^, K^+^ channels^9^,^10^,^11^,^12^,^13^ and excitatory amino acid transporters^14^,^15^. Moreover, klotho counteracts inflammation^16^,^17^. Klotho-hypomorphic mice (*kl/kl*) with defective promoter of the klotho gene suffer from severe tissue calcification, a wide variety of age related disorders and a severely decreased life span^1^,^18^. Conversely, the life span is substantially increased in klotho overexpressing mice^19^. Klotho may similarly influence tissue calcification, ageing and life span of humans^20^,^21^,^22^.Klotho has been implicated in the regulation of depression and cognitive function^23^,^24^,^25^,^26^. Evidence has been presented pointing to an effect of klotho on oligodendrocyte maturation and myelination^27^ and klotho has been postulated to counteract neurodegeneration^28^. Overexpression of klotho has been shown to enhance cognition^23^. Conversely, klotho deficient mice suffer from deterioration of cognitive function^25^,^26^,^29^. The alterations of neuronal function in klotho deficient mice may, however, be due to the severe vascular calcification and may not reflect the effect of klotho or 1,25(OH)_2_D_3_ on cerebral function. 1,25(OH)_2_D_3_ has previously been shown to affect behavior^30^,^31^, emotions and anxiety^32^. In animals, vitamin D deficiency has been shown to decrease explorative behavior and enhance anxiety, aberrant grooming, submissive social behavior, social neglect and maternal cannibalism^33^,^34^,^35^. Prenatal vitamin D deficiency influences murine self-grooming behavior^36^. Deletion of the vitamin D receptor (VDR) has similarly been shown to affect murine behavior^34^,^37^,^38^,^39^,^40^,^41^,^42^. In humans vitamin D deficiency predisposes to several psychiatric disorders, such as depression, bipolar disorder and schizophrenia^32^,^43^,^44^,^45^. The vitamin D receptor (VDR) and vitamin D metabolizing enzymes are expressed widely in cerebral structures including prefrontal cortex, hippocampus, cingulate gyrus, thalamus, hypothalamus, and substantia nigra^46^. VDR gene variants are associated with altered behavior^47^,^48^ as well as susceptibility to age-related changes in cognitive function and depressive symptoms^47^. 1,25(OH)_2_D_3_ serum concentration correlates with extraversion^49^, which is negatively correlated with social phobia, cluster C personality disorders and suicide risk^45^,^50^. Along those lines, the seasonal variations of sun exposure and thus 1,25(OH)_2_D_3_ formation have been associated with seasonal affective disorders^51^,^52^,^53^.

The excessive formation of 1,25(OH)_2_D_3_ in *kl/kl* mice were expected to exert profound effects on behavior. However, due to the severe vascular calcification the *kl/kl* mice are severely ill and not amenable to behavioral studies. Most recent observations revealed that addition of NH_4_Cl into the drinking water fully prevents the severe vascular calcification and rapid ageing of *kl/kl* mice without affecting the excessive formation of 1,25(OH)_2_D_3_ and the increase of plasma phosphate and calcium concentrations^54^. NH_4_Cl is apparently effective by alkalinizing acidic cellular compartments which compromizes the maturation of TGFß, a critical mediator of osteogenic signaling^54^. Aging and life span are almost identical in NH_4_Cl treated *kl/kl*-mice and wild type mice^54^. The NH_4_Cl treated *kl/kl* mice would thus be an ideal model to study the effect of excessive 1,25(OH)_2_D_3_ on behavior. Thus, *kl/kl* mice and wild-type mice were treated with NH_4_Cl (280 mM in drinking water) and with either control diet or vitamin D deficient diet, which has previously been shown to normalize plasma 1,25(OH)_2_D_3_ levels in *kl/kl* mice^55^. The behavior of those mice was explored utilizing open field, dark-light box, O-maze, and forced swimming test.

## Results

**Figure 1:**
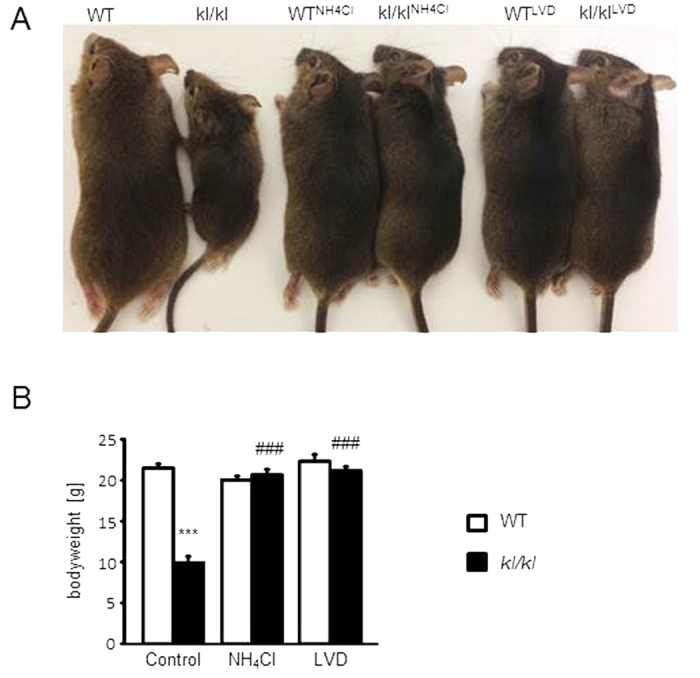
Effect of NH_4_Cl treatment and low vitamin D diet on body weight of wild-type mice and of kl/kl mice. (**A**) Photograph of male wild-type mice (*WT*) as well as male klotho-hypomorphic mice (*kl*/*kl*) without (left) or with NH_4_Cl treatment (15 g/l in drinking water) without (NH_4_Cl, middle) and with (LVD, right) additional low vitamin D diet. (**B**) Arithmetic means ± SEM of body weight (n = 12–30) of wild-type (WT, white bars) and *kl*/*kl* mice (*kl*/*kl*, black bars) either untreated (left bars, Control), treated with NH_4_Cl solution (280 mM in drinking water) (NH_4_Cl, middle bars) or treated with NH_4_Cl and a vitamin D deficient diet (LVD, right bars). ***(p < 0.001) indicates statistically significant differences from respective wild-type mice; ^###^(p < 0.001) indicates statistically significant differences from untreated *kl*/*kl* mice. (ANOVA).

Without NH_4_Cl treatment, klotho-hypomorphic mice (*kl/kl*) suffer from a severe growth deficit (Fig. 1A). Accordingly, the body weight of *kl/kl* mice was significantly lower than the body weight of wild-type mice (Fig. 1B). NH_4_Cl treatment increased significantly the body weight of *kl/kl* mice to similar values as the body weight of wild-type mice (Fig. 1B).

**Figure 2:**
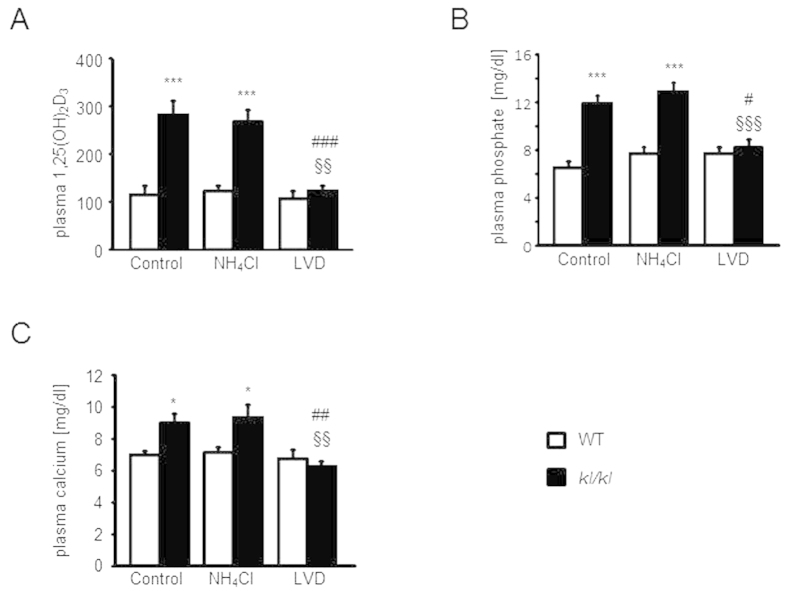
Effect of NH_4_Cl treatment and low vitamin D diet on plasma 1,25(OH)_2_D_3_, phosphate, and Ca^2+^ concentrations of wild-type mice and kl/kl mice. (**A–C**) Arithmetic means ± SEM of (**A**) plasma 1,25(OH)_2_D_3_ (n = 6), (**B**) phosphate (n = 12), and (**C**) Ca^2+^ (n = 12) concentrations of wild-type mice (WT, white bars) and *kl/kl* mice (black bars) either untreated, treated with NH_4_Cl solution (280 mM in drinking water) or treated with NH_4_Cl and vitamin D deficient diet (LVD, right bars). ***(p < 0.001) indicates statistically significant differences from respective wild-type mice (WT); ^#^(p < 0.05), ^##^(p < 0.01), ^###^(p < 0.001) indicates statistically significant differences from untreated *kl*/*kl* mice; ^§§^(p < 0.01), ^§§§^(p < 0.001) indicates statistically significant differences from respective NH_4_Cl treated mice on control diet. (ANOVA).

Plasma 1,25(OH)_2_D_3_ (Fig. 2A), phosphate (Fig. 2B) and Ca^2+^ (Fig. 2C) concentrations were significantly higher in untreated *kl/kl* mice than in wild-type mice, differences not significantly affected by NH_4_Cl treatment. However, vitamin D deficient diet decreased the values of all three parameters in plasma of *kl/kl* mice to values similar as those in wild-type mice.

**Figure 3:**
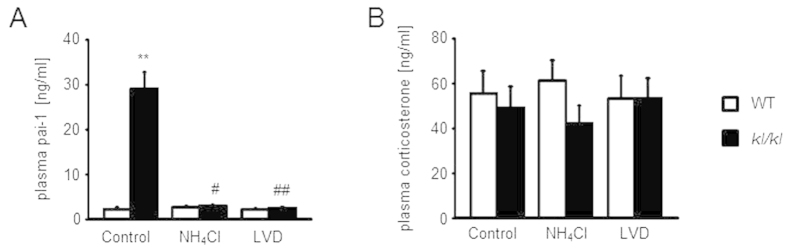
Effect of NH_4_Cl treatment and low vitamin D diet on plasma pai-1and corticosterone levels. (**A**) Arithmetic means ± SEM (n = 8, ♂ = 4, ♀ = 4) of plasma pai-1 concentrations in wild-type mice (WT, white bars) and *kl/kl* mice (black bars) either untreated, treated with NH_4_Cl solution (280 mM in drinking water) or treated with NH_4_Cl and a vitamin D deficient diet (LVD, right bars). *(p < 0.05) indicates statistically significant differences from untreated wild-type mice (Control); ^##^(p < 0.01) indicates statistically significant differences from NH_4_Cl treated *kl*/*kl* mice on control diet. (ANOVA).(**B**) Arithmetic means ± SEM (n = 12, ♂ = 6, ♀ = 6) of plasma corticosterone concentrations of wild-type mice (WT, white bars) and *kl/kl* mice (black bars) either untreated, treated with NH_4_Cl solution (280 mM in drinking water) or treated with NH_4_Cl and a vitamin D deficient diet (LVD, right bars). Blood was drawn between 4 p.m. and 6 p.m.

Plasma Pai-1 levels were assessed as an indicator of aging in all groups. Pai-1 levels in plasma were increased in *kl/kl* mice. NH_4_Cl treatment and the vitamin D deficient diet normalized the plasma Pai-1 levels (Fig. 3A). As an indicator of stress, corticosterone plasma levels were determined. As a result, the plasma corticosterone levels tended to be lower in untreated and NH_4_Cl treated *kl/kl* mice than in the respective wild type mice, differences, however, not reaching statistical significance (Fig. 3B)

Behavioral studies were performed with untreated control wild-type mice (Control), NH_4_Cl treated WT mice and NH_4_Cl treated *kl/kl* mice (NH_4_Cl) under regular diet as well as WT mice and *kl/kl* mice under a vitamin D deficient diet (LVD).

**Figure 4:**
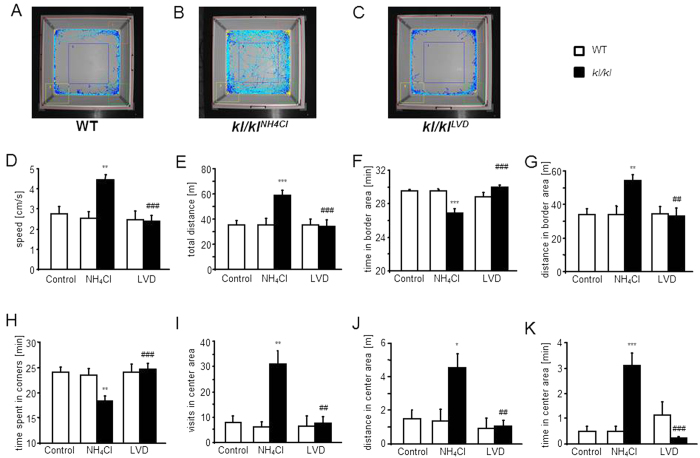
Effect of NH_4_Cl treatment and low vitamin D diet on performance in Open Field Test. (**A–C**) Photographs of the Open Field arena with representative tracings of an untreated, male wild-type mouse (WT) (**A**), a male, klotho-hypomorphic mouse (*kl*/*kl*) treated with 280 mM NH_4_Cl solution (**B**) and a male, NH_4_Cl treated *kl/kl* mouse under vitamin D deficient diet (**C**). (**D–K**) Arithmetic means ± SEM (n = 12–30) of (**D**) average speed measured in the whole observation area, (**E**) total distance travelled during the observation time, (**F**) time spent in the border area of the Open Field arena, (**G**), distance travelled in the border area, (**H**), time spent in the corners of the Open Field arena, (**I**) number of visits in the center area, (**J**) distance travelled in the center area, (**K**) time spent in the center area of wild-type mice (WT, white bars) and *kl*/*kl* mice (*kl*/*kl* black bars) either untreated (Control, left bars), treated with 280 mM NH_4_Cl solution (NH_4_Cl, middle bars) or treated with NH_4_Cl and a vitamin D deficient diet (LVD, right bars). *(p < 0.05), **(p < 0.01), ***(p < 0.001) indicates statistically significant differences from untreated wild-type mice (Control); ^##^(p < 0.01). ^###^(p < 0.001) indicates statistically significant differences from NH_4_Cl treated kl/kl mice on control diet. (ANOVA).

In the open-field, NH_4_Cl treated *kl/kl* mice seemed hyperactive which was already obvious from the recorded tracings (Fig. 4A–C). Computer analysis confirmed the visual impressions revealing significant increases in speed (Fig. 4D) and global distance travelled (Fig. 4E). The NH_4_Cl treated *kl/kl* mice also spent significantly less time in the border area (Fig. 4F) but still travelled larger distances there (Fig. 4G) than wild-type mice. NH_4_Cl treated *kl/kl* mice spent significantly less time in corners (Fig. 4H) and visited the center area more often (Fig. 4I) than wild-type mice. They also travelled larger distances in the center area (Fig. 4J) and spent significantly more time in that section (Fig. 4K). Interestingly, all those behavioral abnormalities were abrogated when *kl/kl* mice were fed a vitamin D deficient diet. There were no differences between untreated wild-type mice and wild-type mice treated with either NH_4_Cl drinking solution or LVD. Rearing behavior is shown in Table 1.

**Figure 5:**
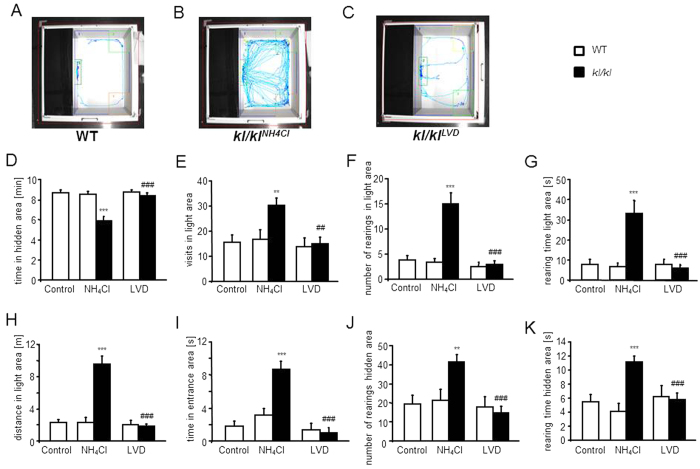
Effect of NH_4_Cl treatment and low vitamin D diet on performance in Light Dark Box. (**A–C**) Photograph of the Light Dark Box with representative tracings of an untreated male wild-type mouse (WT) (**A**), a male, klotho-hypomorphic mouse (*kl*/*kl*) treated with 280 mM NH_4_Cl solution (**B**) and a male, NH_4_Cl treated *kl/kl* mouse under vitamin D deficient diet (**C**). (**D–K**) Arithmetic means ± SEM (n = 12–30) of (**D**) time spent in the hidden area of the Light Dark Box arena, (**E**) number of visits in the light area, (**F**) number of rearings in the light area, (**G**) average rearing time in the light area, (**H**) distance travelled in the light area, (**I**) time spent in the entrance area, (**J**) number of rearings in the hidden area, (**K**) average rearing time in the hidden area of wild-type mice (WT, white bars) and *kl/kl* mice (*kl/kl* black bars) either untreated (Control, left bars), treated with 280 mM NH_4_Cl solution (NH_4_Cl, middle bars) or treated with NH_4_Cl and a vitamin D deficient diet (LVD, right bars). **(p < 0.01), ***(p < 0.001) indicates statistically significant differences from untreated wild-type mice (Control); ^##^(p < 0.01), ^###^(p < 0.001) indicates statistically significant differences from NH_4_Cl treated kl/kl mice. (ANOVA).

**Figure 6:**
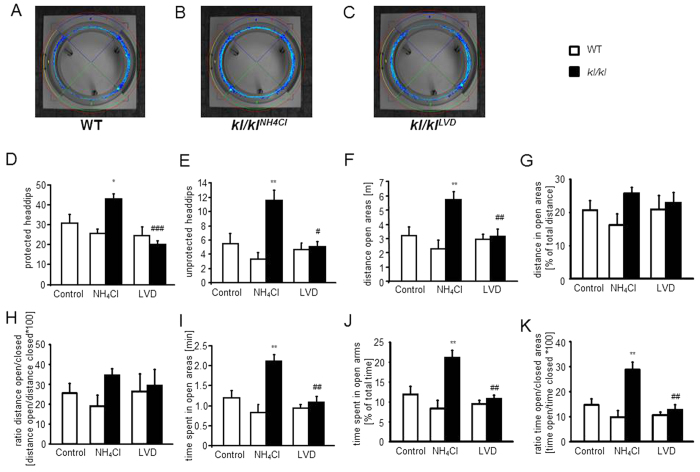
Effect of NH_4_Cl treatment and low vitamin D diet on performance in O-Maze. (**A–C**) Photograph of the O-Maze with representative tracings of an untreated, male wild-type mouse (WT) (**A**), a male klotho-hypomorphic mouse (*kl/kl*) treated with 280 mM NH_4_Cl solution (**B**) and a male, NH_4_Cl treated *kl/kl* mouse under vitamin D deficient diet (**C**). (**D–K**) Arithmetic means ± SEM (n = 12–30) of (**D**) number of protected headdips, (**E**) number of unprotected headdips, (**F**) distance travelled in the open areas, (**G**) distance travelled in the open areas as percentage of total distance, (**H**) ratio of distance travelled in open areas and distance travelled in closed areas, (**I**) time spent in open areas, (**J**) time spent in the open arms as percentage of total time, and (**K**) ratio of time spent in open arms and time spent in closed arms of wild-type mice (WT, white bars) and *kl/kl* mice (*kl/kl* black bars) either untreated (Control, left bar), treated with 280 mM NH_4_Cl solution (NH_4_Cl, middle bars) or treated with NH_4_Cl and a vitamin D deficient diet (LVD, right bars). *(p < 0.05), **(p < 0.01) indicates statistically significant differences from untreated wild-type mice (Control); ^#^(p < 0.05), ^##^(p < 0.01), ^###^(p < 0.001) indicates statistically significant differences from NH_4_Cl treated kl/kl mice. (ANOVA).

The increased activity of NH_4_Cl treated *kl/kl* mice was also apparent in the light dark transition test (Fig. 5A–C). NH_4_Cl treated *kl/kl* mice spent less time in the hidden area (Fig. 5D), visited the light area more often (Fig. 5E), showed more rearings in the light area (Fig. 5F), spent more time rearing in the light area (Fig. 5G), spent more time in the entrance area of the box (Fig. 6H) and travelled larger distances in the light compartment (Fig. 5I). Although NH_4_Cl treated *kl/kl* mice spent less time in the hidden area the number of rearings in the box (Fig. 5J) and the rearing time in the box (Fig. 5K) were significantly increased. Under LVD, *kl/kl* mice performed like wild-type mice. Again neither NH_4_Cl treatment nor LVD had an influence on the behavior of wild-type mice in the light dark transition test. Further parameters are shown in Table 2.

The recorded tracings of the O-Maze test also revealed increased activity in the NH_4_Cl treated *kl/kl* mice (Fig. 6A–C). They showed significantly more protected and unprotected headdips than wild-type mice (Fig. 6D,E). NH_4_Cl treated *kl/kl* mice travelled larger distances in the open areas (Fig. 6F), a differences, however, not reaching statistical significance when normalized to the total distance travelled (Fig. 6G). The ratio between distance travelled in open areas and distance travelled in closed areas tended to be higher in *kl/kl* mice, a difference, however, again not reaching statistical significance (Fig. 6H). NH_4_Cl treated *kl/kl* mice spent more time in the open areas (Fig. 6I), an effect also significant when standardized to the total time spent in the open areas (Fig. 6J). Similarly the ratio of time spent in the open areas and the time spent in closed areas was significantly higher in NH_4_Cl treated *kl/kl* mice (Fig. 6K) as compared to wild-type mice. Treatment with LDV abrogated the abnormal behavioral phenotype of *kl/kl* mice. In the O-Maze test neither NH_4_Cl treatment nor LVD had an influence on the behavior of wild-type mice. Further parameters are shown in Table 3.

**Figure 7:**
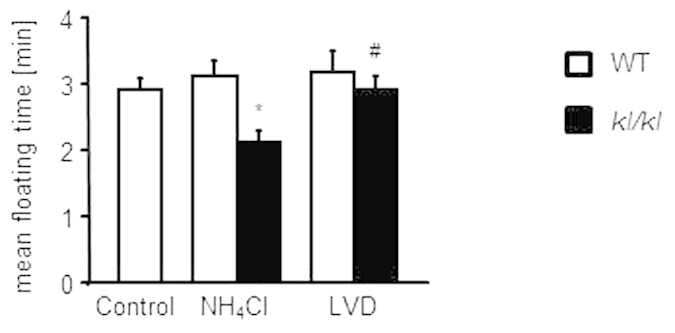
Effect of NH_4_Cl treatment and low vitamin D diet on performance in Forced Swimming Test. Arithmetic means ± SEM (n = 12–30) of floating time of wild-type mice (WT, white bars) and *kl/kl* mice (*kl/kl* black bars) either untreated (Control, left bar), treated with 280 mM NH_4_Cl solution (NH_4_Cl, middle bars) or treated with NH_4_Cl and a vitamin D deficient diet (LVD, right bars). *(p < 0.05) indicates statistically significant differences from untreated wild-type mice (Control); ^#^(p < 0.05) indicates statistically significant differences from NH_4_Cl treated *kl*/*kl* mice. (ANOVA).

In the Forced Swimming Test the NH_4_Cl treated *kl/kl* mice spent significantly less time floating on the surface of the water than wild-type mice (Fig. 7). LVD again abrogated the differences of time floating between *kl/kl* mice and wild-type mice (Fig. 7). Neither of the treatments had an effect on behavior of wild-type mice in the Forced Swimming Test.

Gender differences in the behavioral tests are apparent from Tables 4, 5, 6, 7.

## Discussion

The present observations reveal a dramatic difference between NH_4_Cl treated *kl/kl* mice and NH_4_Cl treated wild-type mice in several behavioral tests measuring exploratory behavior and anxiety. The difference is abrogated by vitamin D deficient diet, indicating that the excessive 1,25(OH)_2_D_3_ formation in *kl/kl* mice accounted for the observed differences between NH_4_Cl treated *kl/kl* mice and wild-type mice. The observations do not rule out more direct effects of klotho deficiency but indicate that the observed differences are in large part explained by excessive formation of 1,25(OH)_2_D_3_. The effects are,however, not necessarily due to a direct effect of 1,25(OH)_2_D_3_ on neuronal function and behavior.

NH_4_Cl treatment had no significant effect in wildtype mice indicating that the NH_4_Cl treatment does not alter any of the measured parameters on its own. Similar to earlier observations^54^, NH_4_Cl treatment did not appreciably influence plasma 1,25(OH)_2_D_3_, Ca^2+^ and phosphate concentrations. NH_4_Cl interferes with osteogenic signaling thus preventing the disastrous tissue calcification in *kl/kl* mice^54^.

The present observations underscore the powerful direct or indirect influence of 1,25(OH)_2_D_3_ on the brain, which presumably accounts for the various cerebral effects of vitamin D deficiency. Decreased serum levels of the 1,25(OH)_2_D_3_ precursor 25(OH)D_3_ were found in patients suffering from depression^56^,^57^. Conversely, vitamin D supplementation has been reported to counteract depressive symptoms^51^,^52^,^53^. Vitamin D deficiency during brain development is apparently a risk factor for the development of schizophrenia, a condition associated with enhanced neuroticism and decreased extraversion^58^. Conversely vitamin D supplementation decreases the risk to develop psychotic-like symptoms^44^.

The present observations did not address the mechanisms underlying the altered behavior of *kl/kl* mice Several mechanisms have been suggested to participate in the cerebral effects of 1,25(OH)_2_D_3_, including antioxidant effects, inhibition of inflammation and vascular injury, stimulation of neurotrophins and improvement of metabolic and cardiovascular function^30^. Vitamin D deficiency has been suggested to modify cellular development, dopamine metabolism, and brain morphology^59^. In theory, 1,25(OH)_2_D_3_ could affect neuronal function by influencing neuronal or glial cytosolic Ca^2+^ activity^60^,^61^,^62^. 1,25(OH)_2_D_3_ may interfere with the cerebral action of glucocorticoids, which are involved in the development of major depression^63^. 1,25(OH)_2_D_3_ dependent calcium binding protein has been observed in nuclei influencing the pineal gland^64^ and vitamin D_3_ deficiency may contribute to the desynchronisation in seasonal affective disorders^65^.

In wild type animals, dietary vitamin D does not necessarily influence 1,25(OH)_2_D_3_ concentration, as 1α-25-hydroxyvitamin D hydroxylase and thus 1,25(OH)_2_D_3_ formation is under tight regulation by FGF23 and klotho^1^. Both, FGF23 and klotho expression are stimulated by 1,25(OH)_2_D_3_ and thus 1,25(OH)_2_D_3_ formation is limited by negative feedback regulation^1^,^66^,^67^. In the presence of klotho and FGF23, the diet becomes critically important only during vitamin D deficiency. The negative feedback is missing in *kl/kl* mice and in those mice the formation of 1,25(OH)_2_D_3_ is a function of dietary vitamin D even at excessive 1,25(OH)_2_D_3_ concentrations. In view of the present observation any regulator of FGF23 and/or klotho expression or any regulator of 1α-25-hydroxyvitamin D hydroxylase may be expected to impact on exploratory behavior. In this respect it is noteworthy that klotho is downregulated and 1,25(OH)_2_D_3_ formation up-regulated by dehydration^68^ and parathyroid hormone^69^, FGF23 is up-regulated and 1,25(OH)_2_D_3_ formation downregulated by lithium^70^,^71^ and 1α-25-hydroxyvitamin D hydroxylase inhibited by CO-releasing molecule CORM-2^72^.

In conclusion, the present observations reveal that disruption of klotho dependent inhibition of 1α-25-hydroxyvitamin D hydroxylase and thus excessive 1,25(OH)_2_D_3_ formation leads to profound stimulation of exploratory behavior.

## Materials and Methods

### Mice

All animal experiments were conducted according to the German law for the welfare of animals and were approved by local authorities (Regierungspräsidium Tübingen). The methods were carried out in accordance with the approved guidelines. The original klotho-hypomorphic (*kl/kl*) mice were generated by Kuro-o *et al*.^19^. In an attempt to insert the rabbit type-I Na^+^/H^+^ exchanger via a standard microinjection method into the genome of the mice, the promoter region of the *klotho* gene was disrupted. The mice do not express the expected transgene but cross-breeding of the heterozygous mice resulted in animals homozygous for the insertional mutation and a severe aging-like phenotype. RT-PCR analysis revealed that *klotho* is still expressed to a low extent and therefore the mice are referred to as klotho-hypomorphic mice. The original *kl/kl* mice had a mixed background of C57BL/6J and C3H/J. Congenic strains of *kl/kl* mice were produced by repeated backcrosses (>9 generations) to the 129Sv inbred strain and used in this study. The mice were generated from heterozygous breedings, and male and female *kl/kl* mice were compared to male and female wild-type (WT) mice^54^. The animals were housed in groups of 2–6 mice per cage. The temperature was set to 22 ± 2 °C and the humidity was 55 ± 10%. The mice had access to either tap water or a solution of NH_4_Cl in tap water (280 mM) ad libitum and were fed either a standard chow diet (Altromin C1000) or a vitamin D deficient diet (Altromin C1017). The lifelong NH_4_Cl treatment started with the mating of the parental generation and was maintained from pregnancy until the end of the experiment. The animals were maintained at a 12:12 h inverted cycle with lights on between 7 p.m. and 7 a.m. Behavioral testing occurred between 7 a.m. and 7 p.m. Only one type of experiment was done on the same day and the home cage rack was brought to the test room at least 30 min before each experiment and dry surfaces of apparatus were thoroughly cleaned with 70% ethanol before releasing the animal. Experiments extended over a total of 4 months, the age was 10–11 weeks at the beginning and 6 months at the end of the experiments. Untreated *kl/kl* mice could not be used in the behavioral tests because of their poor physical condition (Table 8).

### Blood chemistry

Blood specimens were obtained the day after the completion of the behavioral studies between 4–6 p.m. by puncturing the retro-orbital plexus. Plasma phosphate and calcium concentrations were determined utilizing a photometric method (FUJI FDC 3500i, Sysmex, Norsted, Germany). The plasma 1,25(OH)_2_-vitamin D_3_ (IDS, Boldon, UK), corticosterone (DRG, Marburg, Germany) and Pai 1 (Molecular Innovations, Novi, USA) concentrations were measured by ELISA.

### Behavioral studies

For data acquisition, animals were video tracked by the camera 302050-SW-KIT-2-CAM at a resolution of 0.62 to 0.72 pixel (TSE-Systems, Bad Homburg, Germany). Raw data were transferred to Microsoft Excel for further analysis.

Tests were done in the following order: Open-field, light-dark box, O-maze, and forced swimming test. Experiments were performed with diffuse indirect room light produced by dimmable bulbs, adjusted to yield approximately 12 lux in the center of the experimental arena. The only exception was the light-dark-box test where full room light was switched on to obtain approximately 500 lux in the lit chamber. The experiments have been performed as described previously in detail^73^.

For open-field the quadratic open-field arena had a side length of 50 cm, a white plastic floor, and 40 cm high sidewalls made of white polypropylene. Rearing behavior was assessed by a metallic frame surrounding the arena generating a photoelectric barrier (vertical activity). A border area was considered with a width of 10 cm from the wall dividing the arena in a center and a border area. Each subject was released near the wall and observed for 30 min.

For the light-dark box a 40 cm black acryl box was inserted in the open-field arena, which covered 33% of the surface area. An aperture of 10 cm length and 11 cm height with rounded down corners led into the dark box. Each subject was released in the the same corner of the illuminated compartment and observed for 10 min^74^.

For O-maze a 5.5 cm wide annular runway was constructed using grey plastic. It had an outer diameter of 46 cm and was placed inside the above open-field arena 40 cm above the floor^73^,^75^. The two opposing 90° closed sectors were protected by 11 cm high inner and outer walls of grey polyvinyl-chloride, while the remaining two open sectors had no walls. Animals were released in one of the closed sectors and observed for 10 min. Over time, the animal’s exploratory drive competes with their natural avoidance of heights. The mice start to explore the cliff by dipping their heads. As an additional parameter the number of headdips was counted. Differentiated were protected headdips, when the headdips occurred with the mice still in the protected zone, and the unprotected headdips, when the mice left the protected zone completely to explore the cliff. The numbers of headdips were counted manually.

In the forced swimming test mice were placed in a container filled with water of temperatures between 24 and 26 °C. The diameter of the container was 20 cm. The mice were placed in the water without being able to touch the ground. Mice were observed during 6 min and the time they spent without movement, called floating, was recorded^76^.

### Statistics

Data are provided as means ± SEM, *n* represents the number of independent experiments. All data were tested for significance using parametric ANOVA followed by Tukey-Kramer Multiple Comparisons Test in case of equal standard deviations (tested with Bartlett’s) or nonparametric ANOVA (Kruskal-Wallis Test) in case of significant differences in standard deviations followed by Dunn’s Multiple Comparison Test. Only results with *p* < 0.05 were considered statistically significant. The statistical calculations were performed utilizing the Graph Pad Prism software.

## Additional Information

**How to cite this article**: Leibrock, C. B. *et al*. 1,25(OH)_2_D_3_ dependent overt hyperactivity phenotype in klotho-hypomorphic mice. *Sci. Rep.* **6**, 24879; doi: 10.1038/srep24879 (2016).

## Figures and Tables

**Table 1:** Synopsis of rearing parameters in the open field test (arithmetic means ± SEM).

parameter	WT	WT^NH4Cl^	*kl*/*kl*^NH4Cl^	WT^LVD^	*kl/kl*^LVD^	statistics
number of rearings in border area	84.18 ± 14.05	68.87 ± 15.82	138.53 ± 10.70	60.17 ± 13.66	78.10 ± 11.13	P < 0.0001 ANOVA
rearing time in border area [min]	3.03 ± 0.60	2.74 ± 0.68	6.37 ± 0.63	2.56 ± 0.76	2.82 ± 0.50	P < 0.0001 ANOVA
number of rearings in center area	1.36 ± 0.50	1.07 ± 0.93	7.67 ± 1.65	1.42 ± 0.74	0.81 ± 0.45	P < 0.0001 nonparametric ANOVA
Rearing time in center area [s]	1.23 ± 0.48	0.36 ± 0.25	10.88 ± 3.01	0.18 ± 0.12	0.65 ± 0.42	P < 0.0001 nonparametric ANOVA

**Table 2:** Synopsis of behavioral parameters in the Light Dark Box test (arithmetic means ± SEM).

parameter	WT	WT^NH4Cl^	*kl/kl*^NH4Cl^	WT^LVD^	*kl/kl*^LVD^	statistics
time spent in light area [min]	1.28 ± 0.26	1.45 ± 0.28	4.08 ± 0.45	1.26 ± 0.25	1.57 ± 0.25	P < 0.0001 nonparametric ANOVA
average speed [cm/s]	2.43 ± 0.29	2.45 ± 0.24	4.45 ± 0.32	2.48 ± 0.27	2.37 ± 0.29	P < 0.0001 nonparametric ANOVA

**Table 3:** Synopsis of behavioral parameters in the O Maze test (arithmetic means ± SEM).

parameter	WT	WT^NH4Cl^	*kl/kl*^NH4Cl^	WT^LVD^	*kl/kl*^LVD^	statistics
number of visits in open areas	24.27 ± 4.25	20.27 ± 4.86	41.97 ± 5.08	19.73 ± 4.09	23.95 ± 4.22	P = 0.0034 ANOVA
distance in closed areas [m]	12.41 ± 0.82	11.81 ± 0.77	16.58 ± 0.77	11.01 ± 1.35	10.65 ± 0.79	P < 0.0001 ANOVA
total distance [m]	15.45 ± 1.22	14.08 ± 1.15	22.33 ± 1.00	14.02 ± 1.33	13.81 ± 0.96	P < 0.0001 ANOVA
average speed [cm/s]	2.74 ± 0.30	2.37 ± 0.22	3.63 ± 0.18	1.99 ± 0.14	2.40 ± 0.17	P < 0.0001 nonparametric ANOVA

**Table 4:** Differences between male and female mice in the open field test (arithmetic means ± SEM).

parameter		WT	WT^NH4Cl^	*kl/kl*^NH4Cl^	WT^LVD^	*kl/kl*^LVD^
speed [cm/s]	♂	3.00 ± 0.58	2.75 ± 0.39	4.86 ± 0.47	2.37 ± 0.68	2.51 ± 0.40
	♀	2.55 ± 0.41	2.47 ± 0.58	4.11 ± 0.29	2.53 ± 0.64	2.24 ± 0.45
	ttest	0.5266	0.5381	0.1651	0.8619	0.6612
total distance [m]	♂	37.71 ± 6.95	36.03 ± 5.58	61.13 ± 6.53	35.91 ± 7.44	33.81 ± 6.45
	♀	33.09 ± 3.30	32.27 ± 9.82	56.84 ± 5.37	34.74 ± 6.83	35.34 ± 7.33
	ttest	0.5553	0.9000	0.6128	0.9103	0.8762
time in border area [min]	♂	29.66 ± 0.10	29.48 ± 0.40	27.47 ± 0.75	29.28 ± 0.32	29.86 ± 0.05
	♀	29.35 ± 0.39	29.62 ± 0.20	26.43 ± 0.66	28.45 ± 0.99	29.71 ± 0.15
	ttest	0.4611	0.8112	0.3062	0.4423	0.3597
distance in border area [m]	♂	36.31 ± 6.39	34.29 ± 5.93	58.04 ± 5.78	34.40 ± 4.56	33.03 ± 6.28
	♀	34.50 ± 3.45	31.39 ± 9.61	51.21 ± 4.70	34.40 ± 6.77	34.04 ± 6.77
	ttest	0.5153	0.9585	0.3627	1	0.9141
time spent in corners [min]	♂	24.03 ± 1.62	22.33 ± 2.05	18.96 ± 0.88	23.24 ± 2.32	25.41 ± 1.12
	♀	23.97 ± 1.58	24.16 ± 1.61	17.94 ± 1.62	24.91 ± 2.39	23.95 ± 2.13
	ttest	0.9805	0.3798	0.6167	0.6276	0.5417
distance in center area [m]	♂	1.48 ± 0.69	1.74 ± 1.28	5.63 ± 1.32	1.35 ± 1.26	0.77 ± 0.39
	♀	1.60 ± 0.86	0.87 ± 0.48	3.09 ± 0.85	0.49 ± 0.17	1.30 ± 0.68
	ttest	0.8627	0.5802	0.1446	0.5154	0.4969
visits in center area	♂	5.73 ± 1.75	5.13 ± 2.75	23.39 ± 6.39	9.67 ± 7.97	5.82 ± 2.68
	♀	10.18 ± 4.68	7.25 ± 3.44	36.53 ± 8.42	3.00 ± 1.75	9.60 ± 4.74
	ttest	0.3837	0.6284	0.2482	0.4329	0.4854
time in center area [min]	♂	0.35 ± 0.10	0.53 ± 0.40	2.53 ± 0.75	0.72 ± 0.32	0.14 ± 0.05
	♀	0.65 ± 0.39	0.39 ± 0.20	3.57 ± 0.66	1.56 ± 0.99	0.29 ± 0.15
	ttest	0.4611	0.8112	0.3062	0.4423	0.3597
number of rearings in border area	♂	96.73 ± 19.95	68.50 ± 15.56	154.69 ± 15.97	68.50 ± 24.14	86.82 ± 15.65
	♀	71.64 ± 20.00	69.75 ± 30.48	126.18 ± 14.07	51.83 ± 14.50	68.50 ± 16.09
	ttest	0.3850	0.9813	0.1916	0.5671	0.4251
rearing time in border area [min]	♂	3.22 ± 0.81	3.18 ± 1.00	7.56 ± 0.91	2.59 ± 1.14	3.10 ± 0.67
	♀	2.83 ± 0.91	2.33 ± 0.96	6.37 ± 0.63	2.56 ± 1.13	2.52 ± 0.79
	ttest	0.7506	0.5128	0.1016	0.9865	0.5800
number of rearings in center area	♂	1.46 ± 0.78	1.75 ± 1.05	5.69 ± 1.67	1.67 ± 1.31	0.27 ± 0.20
	♀	1.27 ± 0.68	0.25 ± 0.29	9.18 ± 2.59	1.17 ± 0.83	1.4 ± 0.95
	ttest	0.8618	0.4543	0.3019	0.7538	0.2364
rearing time in center area [min]	♂	1.36 ± 0.63	0.28 ± 0.20	6.92 ± 2.05	0.18 ± 0.16	0.34 ± 0.23
	♀	1.10 ± 0.76	0.40 ± 0.36	13.91 ± 5.03	0.18 ± 0.17	0.99 ± 0.86
	ttest	0.7945	0.7266	0.2573	1	0.4501

**Table 5:** Differences between male and female mice in the Light Dark Box test (arithmetic means ± SEM).

parameter		WT	WT^NH4Cl^	*kl/kl*^NH4Cl^	WT^LVD^	*kl/kl*^LVD^
time in hidden area [min]	♂	8.67 ± 0.48	8.71 ± 0.29	6.26 ± 0.57	8.56 ± 0.38	8.47 ± 0.40
	♀	8.79 ± 0.23	8.37 ± 0.53	5.66 ± 0.67	8.91 ± 0.35	8.39 ± 0.32
	ttest	0.8226	0.5645	0.5234	0.5208	0.8785
visits in light area	♂	13.91 ± 3.69	15.75 ± 5.23	26.92 ± 3.69	11.67 ± 3.48	14.36 ± 3.92
	♀	17.10 ± 5.23	17.57 ± 5.75	32.88 ± 4.04	15.67 ± 6.56	15.40 ± 4.16
	ttest	0.6217	0.8180	0.3000	0.6017	0.8579
number of rearings in light area	♂	3.55 ± 1.06	3.63 ± 1.43	16.12 ± 3.32	3.50 ± 1.63	3.00 ± 0.62
	♀	4.27 ± 1.18	3.00 ± 1.07	13.54 ± 2.85	1.67 ± 0.72	3.10 ± 1.34
	ttest	0.6516	0.7377	0.5747	0.3268	0.9449
rearing time light area [s]	♂	8.69 ± 4.40	5.37 ± 2.14	33.31 ± 6.41	5.66 ± 2.86	4.34 ± 1.31
	♀	7.20 ± 2.33	8.51 ± 2.45	33.32 ± 11.67	6.06 ± 2.41	8.13 ± 2.86
	ttest	0.7673	0.3493	0.9985	0.9166	0.2280
distance in light area [m]	♂	2.51 ± 0.55	2.27 ± 0.77	8.870 ± 1.05	1.77 ± 0.57	1.90 ± 0.45
	♀	2.17 ± 0.39	2.33 ± 1.09	10.27 ± 1.58	2.33 ± 0.96	1.84 ± 0.40
	ttest	0.6217	0.9659	0.4444	0.6257	0.9242
time in entrance area [min]	♂	1.55 ± 0.93	3.46 ± 1.17	9.24 ± 1.31	1.39 ± 0.83	1.05 ± 0.75
	♀	1.99 ± 0.03	2.71 ± 1.32	8.25 ± 1.44	1.97 ± 1.66	0.57 ± 0.35
	ttest	0.7532	0.6746	0.6248	0.5077	0.6521
number of rearings in hidden area	♂	21.33 ± 6.29	20.38 ± 7.01	41.35 ± 5.39	16.67 ± 5.10	15.46 ± 5.42
	♀	19.82 ± 6.27	22.86 ± 9.45	41.52 ± 6.01	18.67 ± 10.27	14.00 ± 4.70
	ttest	0.8665	0.8335	0.9444	0.8650	0.8430
rearing time hidden area [s]	♂	5.51 ± 1.07	3.91 ± 1.50	11.19 ± 0.85	6.34 ± 1.82	5.24 ± 1.32
	♀	5.79 ± 1.42	4.41 ± 1.86	11.40 ± 1.07	6.11 ± 2.70	6.34 ± 1.59
	ttest	0.7965	0.8361	0.8345	0.9474	0.5904
time in light area [min]	♂	1.34 ± 0.48	1.29 ± 0.29	3.74 ± 0.57	1.44 ± 0.38	1.53 ± 0.40
	♀	1.22 ± 0.23	1.64 ± 0.53	4.34 ± 0,67	1.09 ± 0.35	1.62 ± 0.32
	ttest	0.8226	0.5645	0.5234	0.5208	0.8785
speed [cm/s]	♂	3.45 ± 0.43	2.41 ± 0.36	4.53 ± 0.38	2.95 ± 0.36	2.23 ± 0.36
	♀	2.40 ± 0.41	2.49 ± 0.33	4.39 ± 0.50	2.03 ± 0.34	2.52 ± 0.48
	ttest	0.9335	0.8677	0.8353	0.0948	0.6244

**Table 6:** Differences between male and female mice in the O Maze test (arithmetic means ± SEM).

parameter		WT	WT^NH4Cl^	*kl/kl*^NH4Cl^	WT^LVD^	*kl/kl*^LVD^
protected headdips	♂	27.00 ± 6.15	23.88 ± 2.86	45.65 ± 3.65	22.83 ± 5.06	20.50 ± 2.66
	♀	34.27 ± 6.42	29.75 ± 3.76	41.00 ± 3.77	26.50 ± 7.21	19.82 ± 2.93
	ttest	0.4231	0.4412	0.4735	0.6858	0.8660
unprotected headdips	♂	4.36 ± 1.50	3.50 ± 1.35	11.77 ± 1.71	5.67 ± 1.45	5.40 ± 1.32
	♀	6.64 ± 2.44	2.75 ± 1.30	11.47 ± 2.14	3.67 ± 1.17	4.73 ± 0.95
	ttest	0.4365	0.8528	0.9350	0.3095	0.6799
visits in open arms	♂	27.27 ± 7.36	18.63 ± 7.10	44.14 ± 5.10	19.50 ± 3.23	22.00 ± 4.69
	♀	21.27 ± 4.46	19.88 ± 7.06	39.59 ± 6.95	20.00 ± 8.74	25.73 ± 7.02
	ttest	0.4937	0.7324	0.5636	0.9552	0.6705
distance in open arms [m]	♂	3.46 ± 1.02	2.24 ± 0.97	6.03 ± 0.75	3.28 ± 0.41	2.98 ± 0.82
	♀	2.96 ± 0.73	2.03 ± 0.82	5.53 ± 0.76	2.57 ± 0.62	3.32 ± 0.66
	ttest	0.6895	0.9564	0.6933	0.3616	0.7462
time in open arms [min]	♂	1.11 ± 0.30	0.73 ± 0.27	2.02 ± 0.26	0.94 ± 0.12	1.12 ± 0.21
	♀	1.27 ± 0.25	0.82 ± 0.33	2.18 ± 0.25	0.96 ± 0.15	1.03 ± 0.24
	ttest	0.6783	0.6649	0.6700	0.9447	0.7722
distance in closed arms [m]	♂	12.42 ± 1.00	12.07 ± 1.03	15.65 ± 0.76	11.69 ± 2.32	10.45 ± 1.26
	♀	12.40 ± 1.34	11.26 ± 1.25	17.92 ± 1.23	10.49 ± 1.56	10.84 ± 1.03
	ttest	0.9898	0.7379	0.3923	0.6770	0.8103
total distance [m]	♂	15.56 ± 1.88	14.30 ± 1.62	21.68 ± 1.22	14.92 ± 2.31	13.42 ± 1.64
	♀	15.35 ± 1.63	13.30 ± 1.76	22.82 ± 1.51	13.12 ± 1.46	14.16 ± 1.13
	ttest	0.9336	0.8454	0.6513	0.5265	0.7114
speed [cm/s]	♂	2.29 ± 0.30	2.22 ± 0.29	3.67 ± 0.20	2.01 ± 0.29	2.47 ± 0.25
	♀	3.82 ± 0.49	2.43 ± 0.34	3.63 ± 0.29	1.97 ± 0.09	2.33 ± 0.23
	ttest	0.1353	0.4781	0.8705	0.8882	0.6823
time in open arms [%]	♂	11.06 ± 2.99	7.34 ± 2.74	20.17 ± 2.55	9.41 ± 1.23	11.22 ± 2.10
	♀	12.70 ± 2.51	8.17 ± 3.33	21.76 ± 2.47	9.55 ± 1.46	10.28 ± 2.39
	ttest	0.6783	0.6649	0.6696	0.9447	0.7721
time open/closed arms *100	♂	13.75 ± 3.94	8.67 ± 3.57	26.89 ± 4.27	10.50 ± 1.54	13.21 ± 2.68
	♀	15.56 ± 3.54	9.85 ± 4.48	30.25 ± 4.88	10.70 ± 1.78	12.35 ± 3.33
	ttest	0.7361	0.6674	0.6428	0.9328	0.8439
distance open arms [%]	♂	22.24 ± 4.33	15.62 ± 5.12	27.81 ± 2.83	22.01 ± 2.73	22.18 ± 4.81
	♀	19.26 ± 3.96	15.29 ± 4.79	24.21 ± 2.24	19.60 ± 8.08	23.45 ± 4.34
	ttest	0.6184	0.7854	0.5286	0.4239	0.6645
distance open/closed arms *100	♂	27.87 ± 6.49	18.52 ± 9.19	38.53 ± 5.27	28.09 ± 4.35	28.50 ± 9.67
	♀	23.86 ± 6.95	18.05 ± 7.11	31.95 ± 4.30	24.52 ± 7.26	30.64 ± 12.81
	ttest	0.6544	0.9178	0.4911	0.2703	0.6927

**Table 7:** Differences between male and female mice in the Forced Swimming test (arithmetic means ± SEM).

parameter		WT	WT^NH4Cl^	*kl/kl*^NH4Cl^	WT^LVD^	*kl/kl*^LVD^
mean floating time [min]	♂	3.10 ± 0.23	3.01 ± 0.33	2.20 ± 0.36	2.93 ± 0.61	2.81 ± 0.30
	♀	2.70 ± 0.30	3.15 ± 0.35	2.02 ± 0.24	3.40 ± 0.29	3.01 ± 0.26
	ttest	0.3018	0.9084	0.6702	0.5035	0.7693

**Table 8:** Number of animals used in the experiment.

	total number of animals	number of ♂	number of ♀
*kl/kl*^NH4Cl^	30	13	17
***kl/kl***^**LVD**^	21	10	11
**WT^NH4Cl^**	15	8	7
**WT**	22	11	11
**WT^LVD^**	12	6	6

## References

[b1] Kuro-o M. Klotho, phosphate and FGF-23 in ageing and disturbed mineral metabolism. Nat Rev Nephrol 9, 650–660 (2013). 23774819 10.1038/nrneph.2013.111

[b2] Topala C. N., Bindels R. J. & Hoenderop J. G. Regulation of the epithelial calcium channel TRPV5 by extracellular factors. Curr Opin Nephrol Hypertens 16, 319–324 (2007). 17565273 10.1097/MNH.0b013e3281c55f02

[b3] Hu M. C. . Klotho: a novel phosphaturic substance acting as an autocrine enzyme in the renal proximal tubule. FASEB J 24, 3438–3450 (2010). 20466874 10.1096/fj.10-154765 PMC2923354

[b4] Dermaku-Sopjani M. . Downregulation of NaPi-IIa and NaPi-IIb Na-coupled phosphate transporters by coexpression of Klotho. Cell Physiol Biochem 28, 251–258 (2011). 21865732 10.1159/000331737

[b5] Imura A. . alpha-Klotho as a regulator of calcium homeostasis. Science 316, 1615–1618 (2007). 17569864 10.1126/science.1135901

[b6] Sopjani M. . Regulation of the Na + /K + ATPase by Klotho. FEBS Lett 585, 1759–1764 (2011). 21605558 10.1016/j.febslet.2011.05.021

[b7] Shumilina E. . Altered regulation of cytosolic Ca(2)(+) concentration in dendritic cells from klotho hypomorphic mice. Am J Physiol Cell Physiol 305, C70–77 (2013). 23596175 10.1152/ajpcell.00355.2012

[b8] Borst O. . 1,25(OH)2 vitamin D3-dependent inhibition of platelet Ca2 + signaling and thrombus formation in klotho-deficient mice. FASEB J 28, 2108–2119 (2014). 24522202 10.1096/fj.13-239277

[b9] Almilaji A. . Upregulation of KCNQ1/KCNE1 K + channels by Klotho. Channels (Austin) 8, 222–229 (2014). 24457979 10.4161/chan.27662 PMC4203751

[b10] Huang C. L. Regulation of ion channels by secreted Klotho. Adv Exp Med Biol 728, 100–106 (2012). 22396165 10.1007/978-1-4614-0887-1_7

[b11] Munoz C. . Klotho sensitivity of the hERG channel. FEBS Lett 587, 1663–1668 (2013). 23603386 10.1016/j.febslet.2013.04.011

[b12] Almilaji A. . Regulation of the voltage gated K channel Kv1.3 by recombinant human klotho protein. Kidney Blood Press Res 39, 609–622 (2014). 25571875 10.1159/000368472

[b13] Almilaji A. . Upregulation of the creatine transporter Slc6A8 by Klotho. Kidney Blood Press Res 39, 516–525 (2014). 25531216 10.1159/000368462

[b14] Almilaji A. . Klotho sensitivity of the neuronal excitatory amino acid transporters EAAT3 and EAAT4. PLos One 8, e70988 (2013). 23923038 10.1371/journal.pone.0070988 PMC3726597

[b15] Warsi J., Abousaab A. & Lang F. Up-Regulation of Excitatory Amino Acid Transporters EAAT1 and EAAT2 by ß-Klotho. Neurosignals 23, 59–70 (2015). 26684854 10.1159/000442604

[b16] Banerjee S. . Klotho ameliorates chemically induced endoplasmic reticulum (ER) stress signaling. Cell Physiol Biochem 31, 659–672 (2013). 23711492 10.1159/000350085

[b17] Izquierdo M. C. . Klotho, phosphate and inflammation/ageing in chronic kidney disease. Nephrol Dial Transplant 27 Suppl 4, iv6–10 (2012). 23258814 10.1093/ndt/gfs426

[b18] Kuro-o M. . Mutation of the mouse klotho gene leads to a syndrome resembling ageing. Nature 390, 45–51 (1997). 9363890 10.1038/36285

[b19] Kurosu H. . Suppression of aging in mice by the hormone Klotho. Science 309, 1829–1833 (2005). 16123266 10.1126/science.1112766 PMC2536606

[b20] Invidia L. . The frequency of Klotho KL-VS polymorphism in a large Italian population, from young subjects to centenarians, suggests the presence of specific time windows for its effect. Biogerontology 11, 67–73 (2010). 19421891 10.1007/s10522-009-9229-z

[b21] Nitta K., Okada K., Yanai M. & Takahashi S. Aging and chronic kidney disease. Kidney Blood Press Res 38, 109–120 (2013). 24642796 10.1159/000355760

[b22] Staude H., Jeske S., Schmitz K., Warncke G. & Fischer D. C. Cardiovascular risk and mineral bone disorder in patients with chronic kidney disease. Kidney Blood Press Res 37, 68–83 (2013). 23548827 10.1159/000343402

[b23] Dubal D. B. . Life extension factor klotho enhances cognition. Cell Rep 7, 1065–1076 (2014). 24813892 10.1016/j.celrep.2014.03.076 PMC4176932

[b24] Gold P. W., Licinio J. & Pavlatou M. G. Pathological parainflammation and endoplasmic reticulum stress in depression: potential translational targets through the CNS insulin, klotho and PPAR-gamma systems. Mol Psychiatry 18, 154–165 (2013). 23183489 10.1038/mp.2012.167 PMC10064987

[b25] Ito K. Frontiers of model animals for neuroscience: two prosperous aging model animals for promoting neuroscience research. Exp Anim 62, 275–280 (2013). 24172191 10.1538/expanim.62.275 PMC4160957

[b26] Park S. J. . Inactivation of JAK2/STAT3 signaling axis and downregulation of M1 mAChR cause cognitive impairment in klotho mutant mice, a genetic model of aging. Neuropsychopharmacology 38, 1426–1437 (2013). 23389690 10.1038/npp.2013.39 PMC3682136

[b27] Chen C. D. . The antiaging protein Klotho enhances oligodendrocyte maturation and myelination of the CNS. J Neurosci 33, 1927–1939 (2013). 23365232 10.1523/JNEUROSCI.2080-12.2013 PMC3711388

[b28] Abraham C. R., Chen C., Cuny G. D., Glicksman M. A. & Zeldich E. Small-molecule Klotho enhancers as novel treatment of neurodegeneration. Future Med Chem 4, 1671–1679 (2012). 22924505 10.4155/fmc.12.134 PMC3564652

[b29] Nagai T. . Cognition impairment in the genetic model of aging klotho gene mutant mice: a role of oxidative stress. FASEB J 17, 50–52 (2003). 12475907 10.1096/fj.02-0448fje

[b30] Cherniack E. P., Troen B. R., Florez H. J., Roos B. A. & Levis S. Some new food for thought: the role of vitamin D in the mental health of older adults. Curr Psychiatry Rep 11, 12–19 (2009). 19187703 10.1007/s11920-009-0003-3

[b31] Schaller M. & Murray D. R. Pathogens, personality, and culture: disease prevalence predicts worldwide variability in sociosexuality, extraversion, and openness to experience. J Pers Soc Psychol 95, 212–221 (2008). 18605861 10.1037/0022-3514.95.1.212

[b32] Minasyan A., Keisala T., Lou Y. R., Kalueff A. V. & Tuohimaa P. Neophobia, sensory and cognitive functions, and hedonic responses in vitamin D receptor mutant mice. J Steroid Biochem Mol Biol 104, 274–280 (2007). 17482806 10.1016/j.jsbmb.2007.03.032

[b33] Kalueff A. V., Lou Y. R., Laaksi I. & Tuohimaa P. Increased anxiety in mice lacking vitamin D receptor gene. Neuroreport 15, 1271–1274 (2004). 15167547 10.1097/01.wnr.0000129370.04248.92

[b34] Kalueff A. V., Lou Y. R., Laaksi I. & Tuohimaa P. Abnormal behavioral organization of grooming in mice lacking the vitamin D receptor gene. J Neurogenet 19, 1–24 (2005). 16076629 10.1080/01677060590949683

[b35] Zou J. . Progressive hearing loss in mice with a mutated vitamin D receptor gene. Audiol Neurootol 13, 219–230 (2008). 18259074 10.1159/000115431

[b36] Burne T. H. . Developmental vitamin D (DVD) deficiency alters pup-retrieval but not isolation-induced pup ultrasonic vocalizations in the rat. Physiol Behav 102, 201–204 (2011). 21059363 10.1016/j.physbeh.2010.11.006

[b37] Burne T. H., Johnston A. N., McGrath J. J. & Mackay-Sim A. Swimming behaviour and post-swimming activity in Vitamin D receptor knockout mice. Brain Res Bull 69, 74–78 (2006). 16464687 10.1016/j.brainresbull.2005.10.014

[b38] Burne T. H., McGrath J. J., Eyles D. W. & Mackay-Sim A. Behavioural characterization of vitamin D receptor knockout mice. Behav Brain Res 157, 299–308 (2005). 15639181 10.1016/j.bbr.2004.07.008

[b39] Kalueff A. V., Lou Y. R., Laaksi I. & Tuohimaa P. Increased grooming behavior in mice lacking vitamin D receptors. Physiol Behav 82, 405–409 (2004). 15276805 10.1016/j.physbeh.2004.04.010

[b40] Kalueff A. V., Lou Y. R., Laaksi I. & Tuohimaa P. Impaired motor performance in mice lacking neurosteroid vitamin D receptors. Brain Res Bull 64, 25–29 (2004). 15275953 10.1016/j.brainresbull.2004.04.015

[b41] Kalueff A. V. . Increased severity of chemically induced seizures in mice with partially deleted Vitamin D receptor gene. Neurosci Lett 394, 69–73 (2006). 16256271 10.1016/j.neulet.2005.10.007

[b42] Sakai S. . Vitamin D receptor signaling enhances locomotive ability in mice. J Bone Miner Res 30, 128–136 (2015). 25043694 10.1002/jbmr.2317

[b43] Glade M. J. Vitamin D: health panacea or false prophet? Nutrition 29, 37–41 (2013). 23085014 10.1016/j.nut.2012.05.010

[b44] Hedelin M. . Dietary intake of fish, omega-3, omega-6 polyunsaturated fatty acids and vitamin D and the prevalence of psychotic-like symptoms in a cohort of 33,000 women from the general population. BMC Psychiatry 10, 38 (2010). 20504323 10.1186/1471-244X-10-38 PMC2889879

[b45] Jylha P. . Differences in neuroticism and extraversion between patients with bipolar I or II and general population subjects or major depressive disorder patients. J Affect Disord 125, 42–52 (2010). 20171742 10.1016/j.jad.2010.01.068

[b46] Eyles D. W., Smith S., Kinobe R., Hewison M. & McGrath J. J. Distribution of the vitamin D receptor and 1 alpha-hydroxylase in human brain. J Chem Neuroanat 29, 21–30 (2005). 15589699 10.1016/j.jchemneu.2004.08.006

[b47] Kuningas M. . VDR gene variants associate with cognitive function and depressive symptoms in old age. Neurobiol Aging 30, 466–473 (2009). 17714831 10.1016/j.neurobiolaging.2007.07.001

[b48] Wrzosek M. . Association between Fok I vitamin D receptor gene (VDR) polymorphism and impulsivity in alcohol-dependent patients. Mol Biol Rep 41, 7223–7228 (2014). 25059118 10.1007/s11033-014-3607-6 PMC4203996

[b49] Ubbenhorst A., Striebich S., Lang F. & Lang U. E. Exploring the relationship between vitamin D and basic personality traits. Psychopharmacology (Berl) 215, 733–737 (2011). 21274699 10.1007/s00213-011-2175-x

[b50] Voracek M. Big five personality factors and suicide rates in the United States: a state-level analysis. Percept Mot Skills 109, 208–212 (2009). 19831101 10.2466/PMS.109.1.208-212

[b51] Bertone-Johnson E. R. Vitamin D and the occurrence of depression: causal association or circumstantial evidence? Nutr Rev 67, 481–492 (2009). 19674344 10.1111/j.1753-4887.2009.00220.x PMC2950608

[b52] Jorde R., Sneve M., Figenschau Y., Svartberg J. & Waterloo K. Effects of vitamin D supplementation on symptoms of depression in overweight and obese subjects: randomized double blind trial. J Intern Med 264, 599–609 (2008). 18793245 10.1111/j.1365-2796.2008.02008.x

[b53] Shipowick C. D., Moore C. B., Corbett C. & Bindler R. Vitamin D and depressive symptoms in women during the winter: a pilot study. Appl Nurs Res 22, 221–225 (2009). 19616172 10.1016/j.apnr.2007.08.001

[b54] Leibrock C. B. . NH4Cl Treatment Prevents Tissue Calcification in Klotho Deficiency. J Am Soc Nephrol 26, 2423–2433 (2015). 25644113 10.1681/ASN.2014030230 PMC4587682

[b55] Fischer S. S. . Hyperaldosteronism in Klotho-deficient mice. Am J Physiol Renal Physiol 299, F1171–F1177 (2010). 20719979 10.1152/ajprenal.00233.2010 PMC3774497

[b56] Hoogendijk W. J. . Depression is associated with decreased 25-hydroxyvitamin D and increased parathyroid hormone levels in older adults. Arch Gen Psychiatry 65, 508–512 (2008). 18458202 10.1001/archpsyc.65.5.508

[b57] Wilkins C. H., Sheline Y. I., Roe C. M., Birge S. J. & Morris J. C. Vitamin D deficiency is associated with low mood and worse cognitive performance in older adults. Am J Geriatr Psychiatry 14, 1032–1040 (2006). 17138809 10.1097/01.JGP.0000240986.74642.7c

[b58] Herran A., Sierra-Biddle D., Cuesta M. J., Sandoya M. & Vazquez-Barquero J. L. Can personality traits help us explain disability in chronic schizophrenia? Psychiatry Clin Neurosci 60, 538–545 (2006). 16958935 10.1111/j.1440-1819.2006.01577.x

[b59] Kesby J. P. . Developmental vitamin D deficiency alters dopamine-mediated behaviors and dopamine transporter function in adult female rats. Psychopharmacology (Berl) 208, 159–168 (2010). 19921153 10.1007/s00213-009-1717-y

[b60] Khanal R. C. & Nemere I. Regulation of intestinal calcium transport. Annu Rev Nutr 28, 179–196 (2008). 18598134 10.1146/annurev.nutr.010308.161202

[b61] Ramasamy I. Recent advances in physiological calcium homeostasis. Clin Chem Lab Med 44, 237–273 (2006). 16519596 10.1515/CCLM.2006.046

[b62] Shumilina E. . Regulation of calcium signaling in dendritic cells by 1,25-dihydroxyvitamin D3. FASEB J 24, 1989–1996 (2010). 20124438 10.1096/fj.09-142265

[b63] Obradovic D., Gronemeyer H., Lutz B. & Rein T. Cross-talk of vitamin D and glucocorticoids in hippocampal cells. J Neurochem 96, 500–509 (2006). 16336217 10.1111/j.1471-4159.2005.03579.x

[b64] Celio M. R. . Monoclonal antibodies directed against the calcium binding protein Calbindin D-28k. Cell Calcium 11, 599–602 (1990). 2285928 10.1016/0143-4160(90)90014-l

[b65] Lansdowne A. T. & Provost S. C. Vitamin D3 enhances mood in healthy subjects during winter. Psychopharmacology (Berl) 135, 319–323 (1998). 9539254 10.1007/s002130050517

[b66] Alesutan I. . 25-hydroxyvitamin d3 1-alpha-hydroxylase-dependent stimulation of renal klotho expression by spironolactone. Kidney Blood Press Res 37, 475–487 (2013). 24247665 10.1159/000355728

[b67] Takenaka T. . Calcitriol Supplementation Improves Endothelium-Dependent Vasodilation in Rat Hypertensive Renal Injury. Kidney Blood Press Res 39, 17–27 (2014). 24821359 10.1159/000355773

[b68] Abed M. . Sensitization of erythrocytes to suicidal erythrocyte death following water deprivation. Kidney Blood Press Res 37, 567–578 (2013). 24335488 10.1159/000355737

[b69] Latus J. . Analysis of alpha-klotho, fibroblast growth factor-, vitamin-D and calcium-sensing receptor in 70 patients with secondary hyperparathyroidism. Kidney Blood Press Res 37, 84–94 (2013). 23552627 10.1159/000343403

[b70] Fakhri H. . Regulation of mineral metabolism by lithium. Pflugers Arch 466, 467–475 (2014). 24013758 10.1007/s00424-013-1340-y

[b71] Zhang B. . Lithium- Sensitive Store-Operated Ca2 + Entry in the Regulation of FGF23 Release. Neurosignals 23, 34–48 (2015). 26674092 10.1159/000442602

[b72] Feger M. . Effect of carbon monoxide donor CORM-2 on vitamin D3 metabolism. Kidney Blood Press Res 37, 496–505 (2013). 24247848 10.1159/000355730

[b73] Lang U. E. . Reduced locomotion in the serum and glucocorticoid inducible kinase 3 knock out mouse. Behav Brain Res 167, 75–86 (2006). 16246437 10.1016/j.bbr.2005.08.017

[b74] Crawley J. & Goodwin F. K. Preliminary report of a simple animal behavior model for the anxiolytic effects of benzodiazepines. Pharmacol Biochem Behav 13, 167–170 (1980). 6106204 10.1016/0091-3057(80)90067-2

[b75] Konig M. . Pain responses, anxiety and aggression in mice deficient in pre-proenkephalin. Nature 383, 535–538 (1996). 8849726 10.1038/383535a0

[b76] Leibrock C. . Akt2 deficiency is associated with anxiety and depressive behavior in mice. Cell Physiol Biochem 32, 766–777 (2013). 24080829 10.1159/000354478

